# Rib Mediated Non-Cardiac Chest Pain: A Case Report

**DOI:** 10.7759/cureus.10831

**Published:** 2020-10-06

**Authors:** David P Newman, Brittany J Jansen, Alexandra Scozzafava, Ryan Smith, Brian C Mclean

**Affiliations:** 1 Interdisciplinary Pain Management Clinic, Tripler Army Medical Center, Honolulu, USA; 2 Physical Therapy, Naval Health Clinic Hawaii, Honolulu, USA; 3 Physical Therapy, Irwin Army Community Hospital, Fort Riley, USA; 4 Cardiology, Adventist Health System, Kailua, USA; 5 Pain Management, Landstuhl Regional Medical Center, Landstuhl, DEU

**Keywords:** non-cardiac chest pain, rib, musculoskeletal manipulation

## Abstract

Non-cardiac chest pain (NCCP) is a very common and functionally limiting pain complaint that vexes patients and medical providers leading to time-consuming and expensive diagnostic work-ups as well as significant disability and lost productivity. Despite extensive debate and research, there is no definitive treatment recommendation or high-level evidence to support a conservative care treatment approach, or interventional management procedures for the diagnosis and alleviation of NCCP. In patients presenting with chest pain, after ruling out life-threatening causes, the diagnosis of NCCP is made. This process is a diagnosis of exclusion rather than a specific etiology with a defined treatment plan. This results in specialty consultation, advanced diagnostic testing, and delayed definitive care. A better triage process may include the incorporation of diagnostic maneuvers at the primary care and emergency room to justify referral to a musculoskeletal specialist in lieu of or during advanced diagnostic work-up. After the diagnosis of NCCP is made in our young and active patient population, we have seen significant success in the application of manipulation and a functional restoration program similar to the presented case. To our knowledge, this treatment approach has not been previously described. While this management strategy may be taught in physiotherapy courses, we provide the case to illustrate a multimodal treatment approach that seems to be unknown or underutilized based on the number of referrals and prevalence of this condition.

## Introduction

Non-cardiac chest pain (NCCP) is a common complaint in both the emergency department (ED) and primary care settings. Approximately 55% of chest pain cases presenting to the ED [1] and more than 80% of cases in the primary care settings [2] are diagnosed as NCCP. Considering this diagnosis is typically present in a low-risk group of patients and the non-specific nature of these symptoms, a broad differential diagnosis must be considered as potential causes of the chest pain. Musculoskeletal, gastrointestinal, psychogenic, and pulmonary conditions are all considered as potential causes of NCCP [2]. In many cases, these patients undergo an extensive cardiopulmonary evaluation to include electrocardiogram, echocardiogram, stress testing, CT scans, and pulmonary function testing without providing the patient with a definitive diagnosis for their present symptoms. 

Differentiating the diagnosis of chest pain begins with an initial work-up to exclude acute, life-threatening etiologies. Even in the setting of an initial negative cardiac evaluation, approximately 16% of patients undergo advanced medical imaging [3]. Upon discharge from the ED or primary care office, patients are sent for further extensive cardiac, pulmonary, and/or gastroesophageal testing despite 45% of patients’ discharge diagnoses as being musculoskeletal chest pain. Only 1.5% of those patients are referred to a musculoskeletal specialist upon discharge [4]. This current process results in delayed decisive care and increased cost to the health care system, without relief of the patient’s symptoms. 

After an initial negative cardiac work-up is performed, the involvement of a musculoskeletal specialist (i.e. physical therapist, chiropractor, or osteopath) to perform low-risk manual evaluation to assist in ruling in musculoskeletal causes as part of the differential of NCCP would assist in both diagnosis and definitive treatment. The purpose of this case study is to describe the application of a multimodal musculoskeletal rehabilitation program for the treatment of patients with rib mediated NCCP and make recommendations to modify current diagnostic algorithms to mitigate unnecessary costs, advanced imaging, and delayed care.

## Case presentation

A 24-year-old male, active duty U.S. Marine presented with an eight-month history of sternal pain. The pain was primarily left sided to include the costochondral cartilage, anteriorly. The patient’s symptoms were insidious in onset with no history of trauma. At the time of occurrence, the patient was in an advanced training program that involved daily periods of swimming with push-ups at the side of a 50-meter pool between laps. The initial onset of chest pain occurred in the evening while he was lying in bed. The patient was seen in the emergency department (ED) with 5/10 pain described as central pressure and left-sided tightness. Associated symptoms included dyspnea, left arm pain, finger numbness and dizziness. The patient reported similar episodes over the previous years which was attributed to a history of anxiety. He also reported a vague cardiac history as an infant, but an echocardiogram (EKG) in high school was normal. His twin brother died as an infant from heart failure. The patient uses electronic cigarettes and consumes alcohol occasionally (approximately four to six drinks intermittently on the weekend). The patient had returned from a nine-month deployment to Korea one month prior to this episode. The week prior to the onset of chest pain, the patient had been seen in the ED for left-sided mid-back pain at the T8 region and treated with ketorolac with resolution of his pain the following day. 

Upon examination, his vital signs were assessed (Table 1). Blood tests, a 12-lead EKG, and radiographs were performed. The EKG was interpreted as normal sinus rhythm, PR interval, QRS complexes, and STs and T waves. The chest x-ray was normal. The laboratory values for lipase and troponin-I cardiac were 19 Units/L (reference range: 8-78) and less than 0.010 ng/mL (reference range: 0-0.034), respectively. He was treated with 400 mg ibuprofen orally every six hours as needed for pain and discharged from the ED. 

**Table 1:** Patient vital signs

	Results
Blood pressure	131/64
Heart rate	68 beats/minute
Respiration rate	18/minute
Oxygen saturation	100% on room air
Temperature	98.1 F

The patient was referred to the Cardiology Clinic and seen the next day. An extensive cardiac workup was performed, to include coronary computed tomography angiogram and repeat EKG, which were normal. The patient was subsequently referred to the Pulmonary Clinic for persistent/recurrent shortness of breath. Pulmonary functional testing, chest x-ray, laboratory tests, and a methacholine challenge test were negative.

After 12 months of recalcitrant sternal pain, the patient was referred to the Interdisciplinary Pain Management Clinic (IPMC) at Tripler Army Medical Center, Honolulu, Hawaii. The patient’s primary goal was to find out what was wrong. His secondary goal was to remain in the Marine Corps.

Physical examination

**Figure 1:**
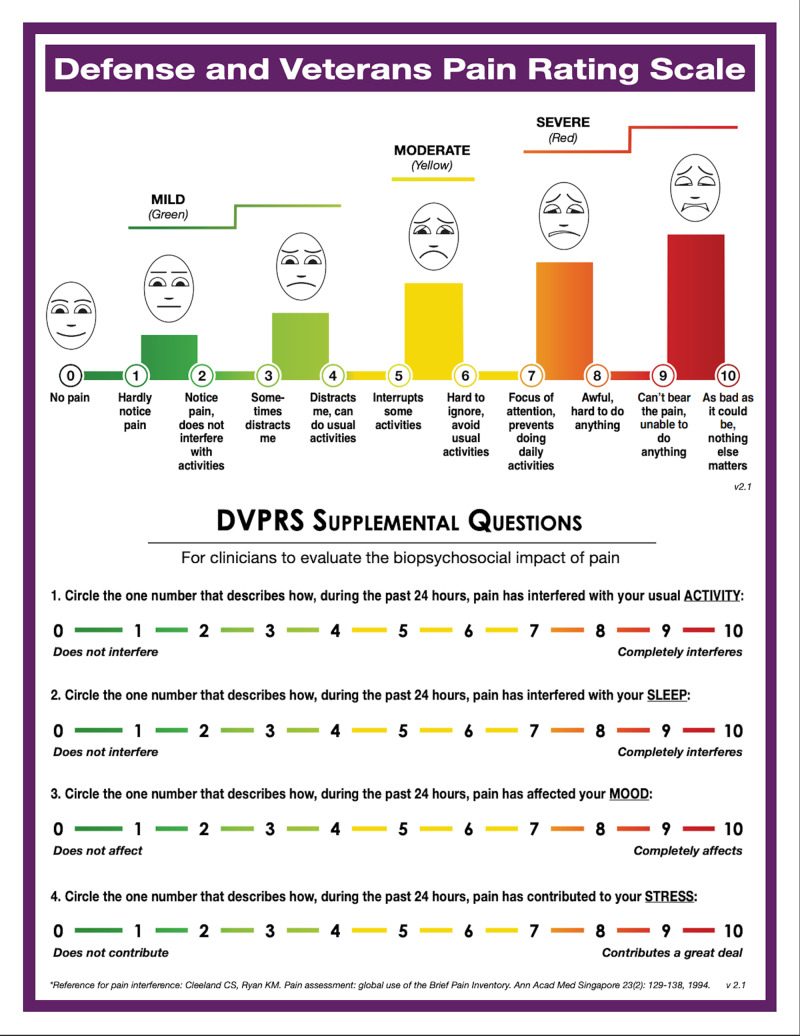
Defense and Veterans Pain Rating Scale (DVPRS)

Physical evaluation at the IPMC revealed that the patient’s pain was localized to the distal half of the left lateral sternal border. The patient completed a Defense and Veterans Pain Rating Scale (DVPRS) (Figure 1) [5] with his pain level of 4/10. The subsets of the pain supplemental questions for activity, sleep, mood, and stress were rated at 4/10, 5/10, 4/10, and 5/10, respectively and were assessed at each visit (Table 2). The pain quality was described as dull and constant. All movements requiring contraction of the pectoral muscles reproduced pain; however, the most specific pain provoking activities were push-ups, upper body weightlifting in the gym, jogging, and swimming.

**Table 2:** Defense and Veterans Pain Rating Scale (DVPRS) per visit

	First Visit	Second Visit	Third Visit	Fourth Visit	Fifth Visit	Sixth Visit	Seventh Visit	Eighth Visit	Ninth Visit
Current Pain Level	4	4	6	6	8	3	3	2	0
Pain with Activity	4	2	7	5	7	3	3	2	0
Pain Affecting Sleep	5	4	5	5	9	6	3	2	0
Pain Affecting Mood	4	4	5	4	7	3	3	2	0
Pain Contributing to Stress	5	4	7	4	7	3	3	2	0

Upon observation, the patient demonstrated a forward head and shoulder posture. He was highly guarded with thoracic extension and with chest expansion during inspiration. Cervical spine screening to include range of motion (ROM) assessment, Spurling’s test for nerve root compression, and intervertebral joint motion testing with passive side glides in supine at C3 through C7 were all negative. Thoracic ROM was assessed grossly. He reported pain with extension greater than flexion. Side bending to the left was painful and worsened with maximal inhalation. There was no change in pain with thoracic rotation.

Motion palpation testing

Segmental vertebral and rib motion assessment was performed utilizing several techniques starting at the cervicothoracic junction and moving inferiorly. Assessment of the first rib hypomobility was performed using the cervical lateral flexion rotation test [6]. The test is performed in sitting with the head passively moved into rotation and then laterally flexed. The amount of flexion is compared to the contralateral side with a positive test indicated by a decrease in flexion.

**Figure 2:**
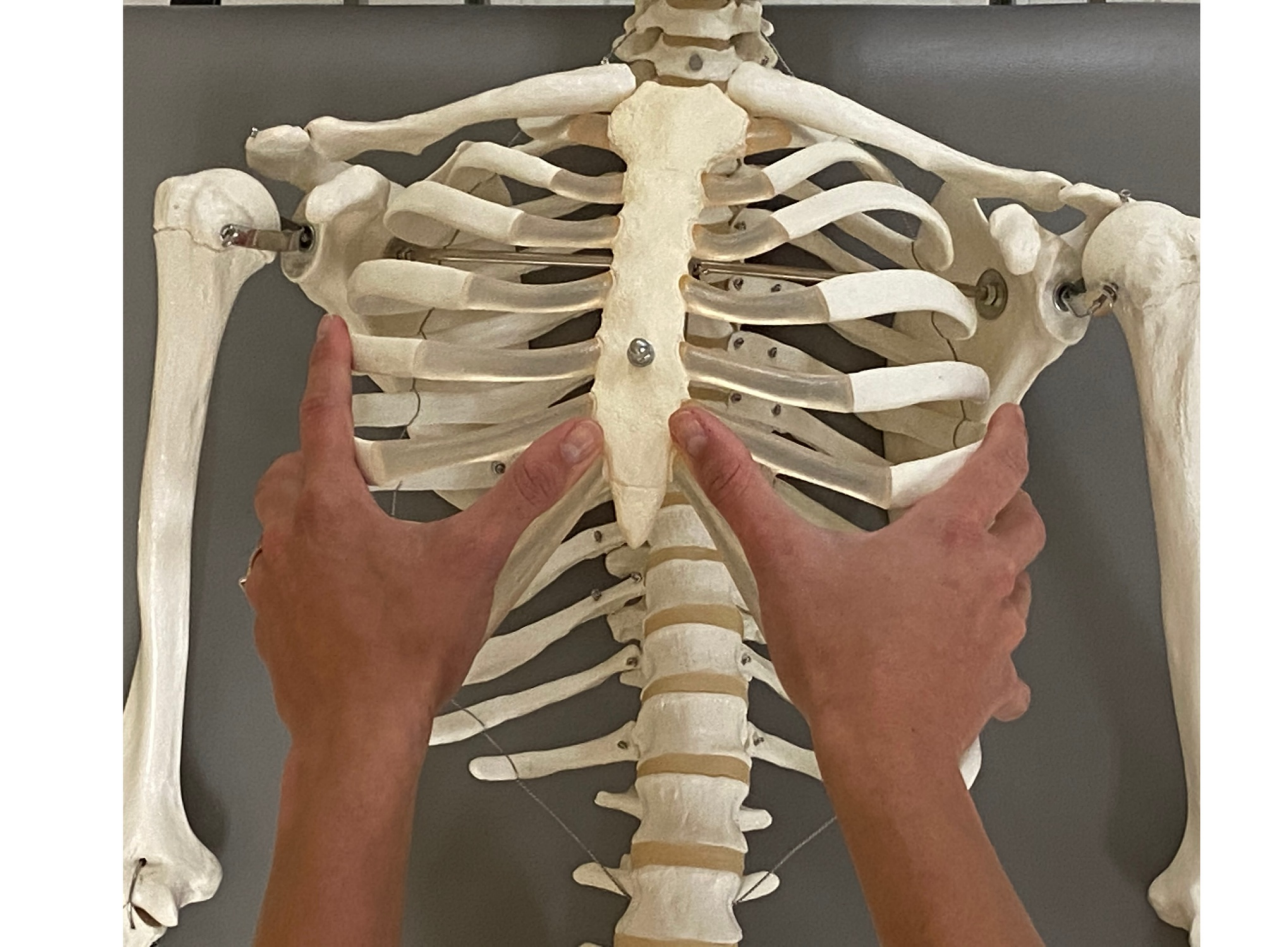
Assessment of seventh rib excursion - hand placement on a skeleton. (Photograph: Scozzafava, AM. Rib Excursion Assessment Technique - Skeleton. Reproduced with permission of author; 2020).

**Figure 3:**
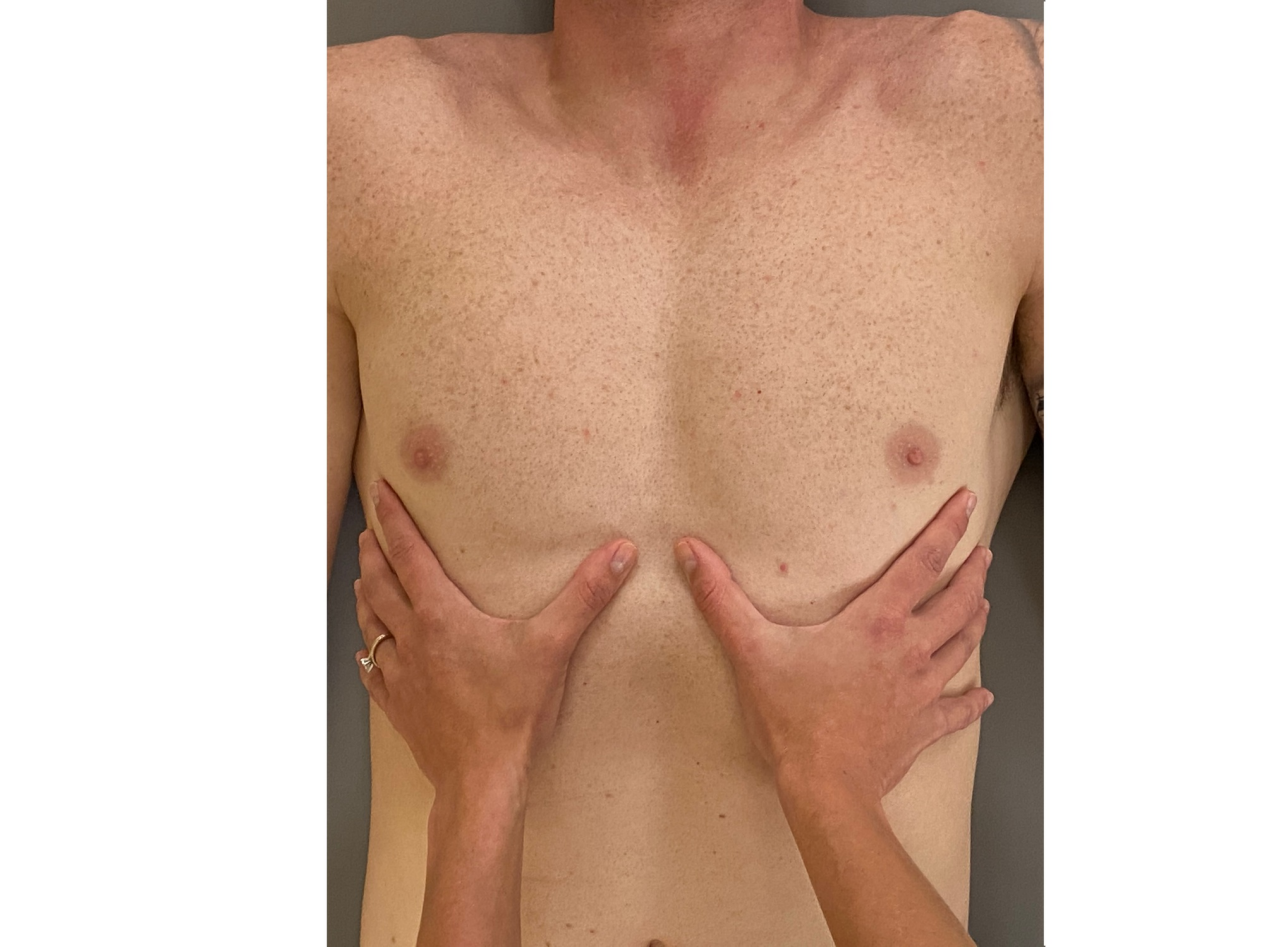
Assessment of seventh rib excursion - while the fingers are place on bilateral seventh ribs, the patient inhales deeply. Asymmetric motion indicates potential rib hypomobility. (Photograph: Scozzafava, AM. Assessment of 7th Rib Excursion Technique - Patient.  Reproduced with permission of author; 2020).

In supine, excursion of the ribs was assessed both visually and through palpation of the rib during inhalation (Figures 2, 3). This osteopathic technique, which assesses for asymmetric motion of the ribs during breathing, was selected due to its diagnostic utility demonstrated in a case series on painful rib syndrome [7]. Movement of the left seventh rib was delayed as compared to the right side.

**Figure 4:**
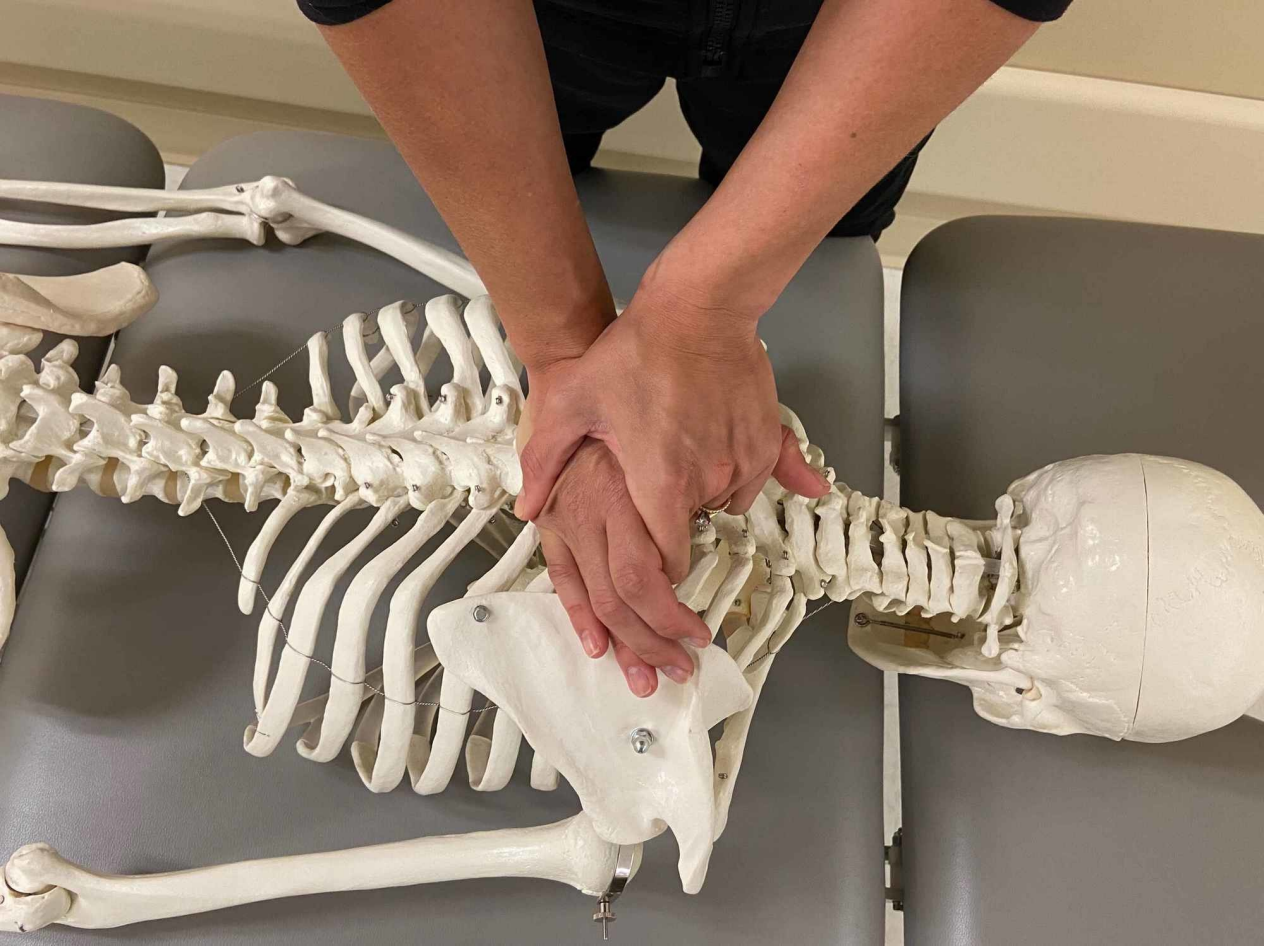
Passive accessory intervertebral motion testing – hand placement on a skeleton. (Photograph: Scozzafava, AM.  Passive Accessory Intervertebral Motion Testing – Skeleton.  Reproduced by permission of author; 2020).

**Figure 5:**
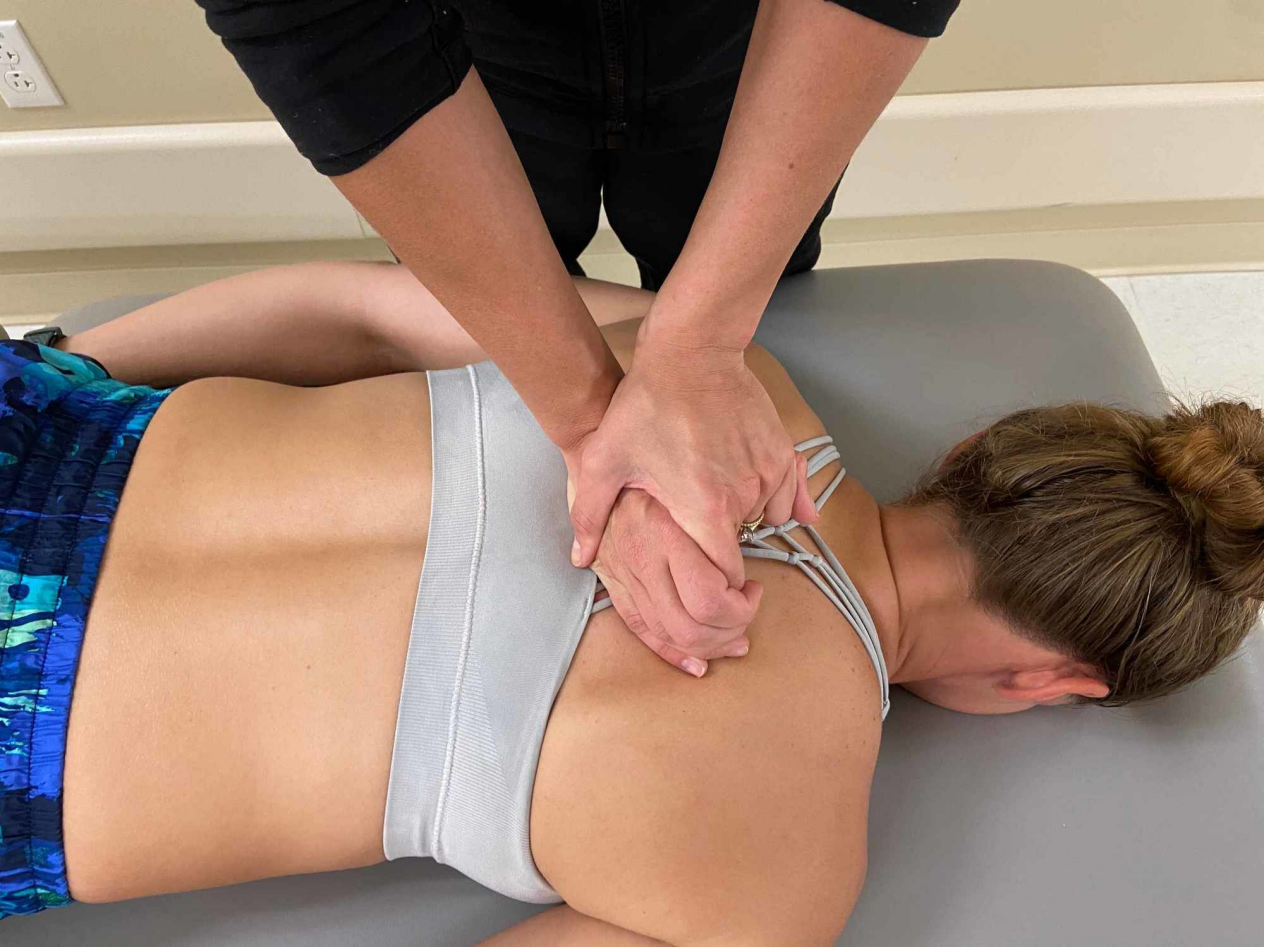
Passive accessory intervertebral motion testing – technique applied to a patient. Pressure is applied to the T7 spinous process downwards assessing for restricted motion or pain reproduction. (Photograph: Scozzafava, AM.  Passive Accessory Intervertebral Motion Testing - Patient. Reproduced by permission of author; 2020).

Passive accessory intervertebral motion (PAIVM) testing was performed in prone to the T1 through T12 spinous processes. The technique is commonly utilized by physical therapists and is considered to be valid in assessing segmental hypomobility [8]. Intra-rater and inter-rater reliability in assessment of joint mobility in the thoracic spine and ribs is good to moderate, respectively [9]. Decreased motion was assessed at T4/5 and T7/8 with pain reproduction anteriorly along the sternum when pressure was applied to the T7 spinous process (Figures 4, 5).

Strength and flexibility assessment

Flexibility and strength assessments were made in supine. The scalenes were tight bilaterally with considerable muscle length deficits on the left side as compared to the right. The pectoralis minor was tight bilaterally. The pectoralis major was assessed with manual resistance. The patient could not generate much force due to worsening of his sternal pain on the left side.

Intervention

**Figure 6:**
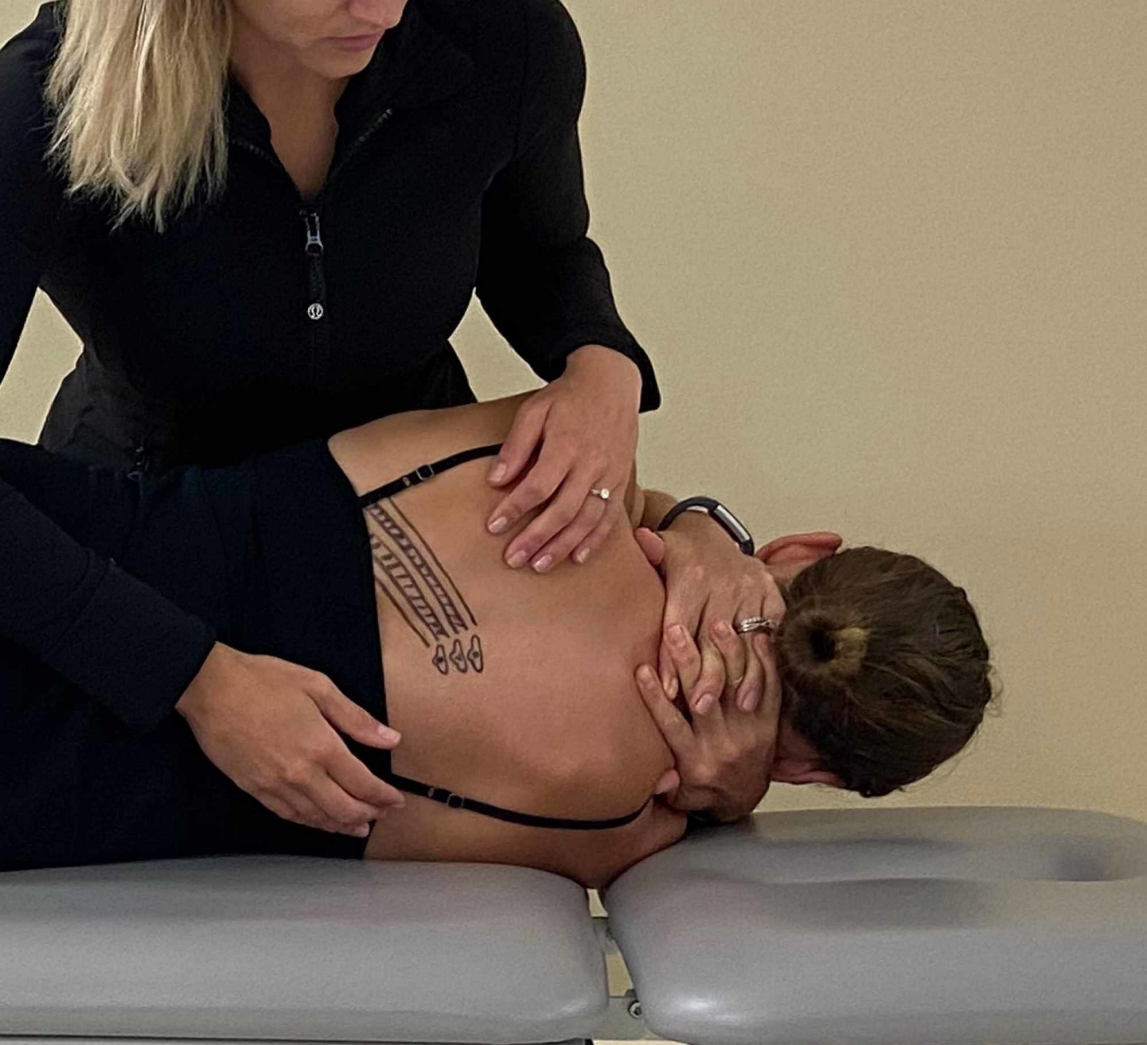
Patient positioning – with the patient positioned in is side lying, the location of the hypomobile rib is identified. In this example, the sixth through eighth ribs are drawn with intercostal muscles visualized by striped lines. (Photograph: Scozzafava, AM. Osteopathic Manipulation Technique – Patient Positioning. Reproduced by permission of author; 2020).

**Figure 7:**
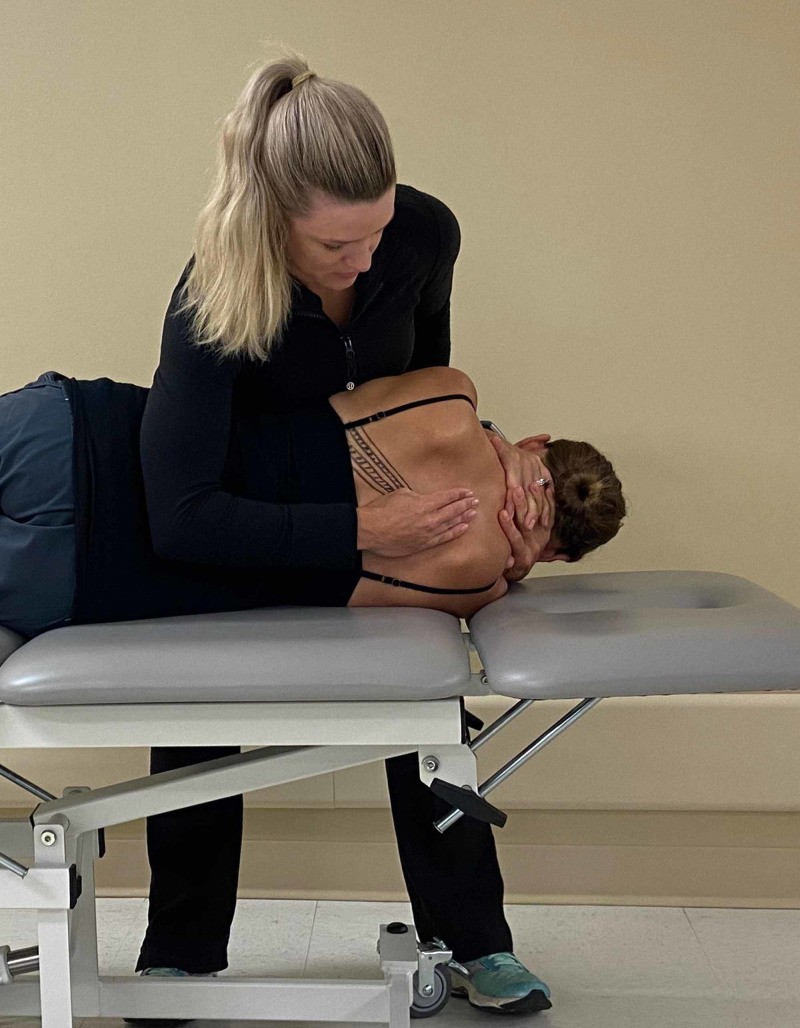
Hand positioning – the provider’s first metacarpal phalangeal joint of the palpating hand is placed over the seventh rib at the costotransverse joint and slid inferiorly to induce posterior rotation to the rib. (Photograph: Scozzafava, AM. Osteopathic Manipulation Technique – Hand Positioning. Reproduced by permission of author; 2020).

**Figure 8:**
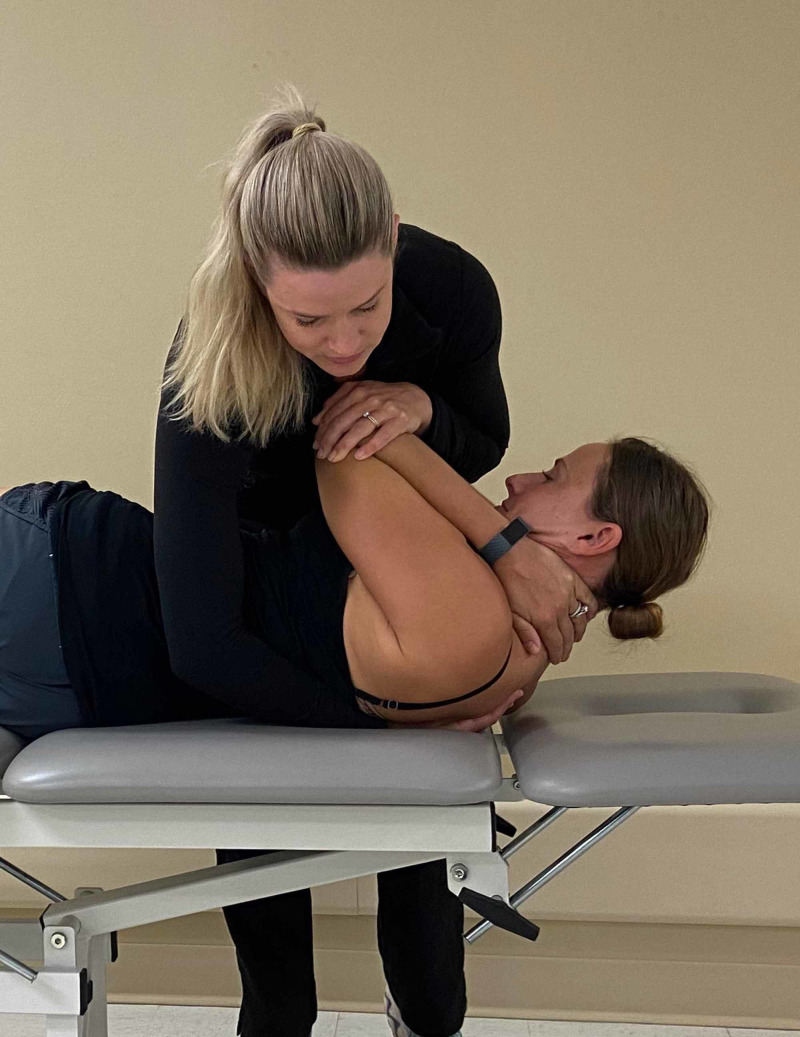
Manipulation technique – the provider moves the patient into supine position. While the patient exhales, the provider and imparts a high velocity, low amplitude force through the patient’s arms towards the examiner’s hand. (Photograph: Scozzafava, AM. Osteopathic Manipulation Technique - Thrust Maneuver. Reproduced by permission of author; 2020).

The patient was treated upon the initial evaluation with osteopathic manipulation techniques (OMT). OMT or high-velocity, low-amplitude (HVLA) techniques have been shown to be effective in the treatment of rib dysfunction [10] and segmental hypomobility in the cervical and thoracic spine in patients with cervicothoracic angina [11]. The segmental hypomobility in the thoracic spine was treated with anterior to posterior force applied into thoracic extension at T4/5 and T7/8 with audible cavitation. No pain was reproduced with PAIVM testing after treatment. A posterior rotation force was applied to the left 7th rib with audible cavitation (Figures 6-8). Upon performing active thoracic ROM, there was a 50% reduction in pain with side bending and inspiration.

Manipulative therapy was followed by soft tissue mobilization and manual stretching applied to the left pectoralis minor. He was instructed in a home exercise program consisting of pectoralis minor stretching and strengthening of the serratus anterior and lower trapezius designed to address tissue tightness, tissue weakness, and correct for postural changes [12]. The pectoralis minor was stretched with the patient's elbow placed on the wall of a doorway at 60 degrees of abduction. He was asked to move his trunk anteriorly until a stretch was felt. The scalenes were stretched in standing by pulling the skin along the jaw superiorly with the ipsilateral hand holding onto the underside of a desk as a counterforce. Each stretch was held for 30 seconds and repeated two to three times per day. The serratus anterior was strengthened by performing a reverse dip on a chair. The contraction was held for three to five seconds and repeated until fatigue. The lower trapezius was addressed using elastic resistance bands. He performed each strengthening exercise for three sets of 10 reps every other day. He was asked to follow-up two days later for reassessment and to develop his functional goals.

Upon follow-up, the patient reported a 70% relief of symptoms after the initial treatment. He reported experiencing most of his relief for the first two hours following treatment. Later in the evening, he noted pain along the superior portion of his sternum with sitting. The stretching program was more helpful in mitigating his pain than the strengthening exercises; however, he had only performed one set of the strengthening exercises and the recommended frequency was every other day. The physical examination and segmental motion testing were repeated, and hypomobility was noted at the T4/5 segment. The T7/8 segment and seventh rib was non-painful and no longer hypomobile. Pain was no longer reported at the distal half of the sternum. The pain was now noted along the mid sternum anteriorly and at the T4/5 segment posteriorly to palpation and motion palpation testing.

The manual and manipulative therapy described above was repeated. Pectoralis minor tightness was addressed manually utilizing an active release method. With the patient supine, the arm is abducted passively to 90 degrees. The pectoralis minor is assessed by following the rib cage with the fingers. Pressure is applied to the pectoralis minor in a medial direction while the patient actively adducts the arm. This was repeated several times and his sternal pain decreased from a 4/10 to a 1/10. His new functional goals were to return to throwing in four to six weeks and swimming without pain in six to 12 weeks.

At the third through fifth visits, his pain pattern changed. The patient reported pain along the manubrium especially with attempts at performing planks or push-ups as part of his unit physical training program that he was still trying to participate in. Upon examination, the first rib was elevated bilaterally via positive cervical lateral flexion rotation test. Contraction of the pectoralis major reproduced his pain. There was a palpable delay in left-sided rhomboid activity during active shoulder abduction resulting in scapular dyskinesia [13].

Following OMT to the first rib [14], the patient reported no pain along the manubrium at rest or during the performance of push-ups immediately following OMT. To address the rhomboids, directional cupping [15] was applied to area above the muscle for a period of five minutes. There was symmetrical scapular motion with active shoulder abduction after treatment. Given the motion fault at the first ribs, scalene stretching was emphasized as part of his exercise program. The patient was treated in the clinic twice a week for this two-week period.

On the sixth visit, the patient reported that two days prior, he awoke in the morning and his upper central chest was bright red and painful. The patient self-treated with ice and non-steroidal anti-inflammatory (NSAID) medication which did resolve the symptoms by the next day. The patient reported continued pain along the manubrium, but his pain level was 3/10, which was a 50% decrease from the previous visit (Figure 3). As with the previous treatment, OMT to the first ribs resulted in immediate and complete pain resolution. The patient was instructed to continue his course of NSAIDS for 14 days.

Over the next two visits, he continued to report soreness along the manubrium that was well-controlled with NSAIDs. His motion palpation testing was negative for hypomobility or pain provocation. His exercise program was progressed to eccentric pectoralis major strengthening. Upon the ninth and final visit, the patient reported no pain with return to upper body weightlifting, throwing, and running. The patient had met his functional goals early and was subsequently discharged from care. He was contacted two months following discharge to determine his status. The patient reported that he was pain-free and was fully engaged in normal duty and physical training activities.

## Discussion

Non-cardiac chest pain can be difficult to diagnose given the overlap in symptoms from pulmonary, gastroesophageal, musculoskeletal chest wall, and psychiatric conditions [16]. The musculoskeletal chest pain differential includes intercostal myalgia, costochondritis, Tietze syndrome, slipping rib syndrome, costovertebral dysfunction, thoracic outlet syndrome, and segmental thoracic dysfunction [17]. Conservative management of patients with rib mediated non-cardiac chest pain can be provided via a multi-modal rehabilitation program designed to correct biomechanical faults at the costovertebral joints, supporting muscles at the sternum as well as ribs, and tendinous attachments at the sternum and/or costochondral cartilage. This case study demonstrates the successful application of manual and manipulative therapy with soft tissue mobilization techniques and exercise to a patient with chronic musculoskeletal chest pain.

Prior to any musculoskeletal assessment, it is critical to rule out potential cardiac pathology. As described above, the patient did undergo initial testing via a 12-lead EKG and assessment of cardiac troponin upon presentation to the ED. High-sensitivity cardiac troponin assays have been shown to have high diagnostic accuracy for ruling in and out acute myocardial infarctions [18]. Despite normal values for the initial tests, this patient did undergo further cardiac as well as pulmonary work-ups over a several month period.

Given the high costs and delayed definitive care of the work-up for non-cardiac chest pain [16], we would argue that patients whose initial cardiac testing (i.e. normal 12-lead ECKG and normal values for cardiac troponin) is negative, should undergo palpatory assessment of the ribs during inhalation and provocation testing along the costovertebral joints by the emergency room physician. Musculoskeletal mediated pain is referred to the sternum due to abnormal firing of nociceptive impulses from surrounding muscles impacting the collateral branches of the intercostal nerve that innervates the costovertebral and costotransverse articulations [19]. The likelihood that the pain is musculoskeletal in nature increases when pain is reproducible by palpation or if there is a decrease in pain with pain medications [20]. 

If positive findings are demonstrated upon exam, the patient can then be referred to physical therapy, a chiropractor, or osteopath to perform low-risk manual evaluation to assist in ruling in musculoskeletal causes as part of the differential. This referral can be done concurrent with referral to Cardiology for more advanced diagnostics thereby saving time and potentially mitigating unnecessary tests such as an echocardiogram, cardiac MRI, or CT angiography if the patient is otherwise healthy and has no cardiac risk factors. Given the time delay in scheduling of these more advanced diagnostic procedures, early diagnosis of potential musculoskeletal etiology could defer the potential financial costs and patient uncertainty. In this case, definitive treatment was provided through a relatively low number of clinic visits over a one-month period.

Although our case report provides important information about various causes and treatments of non-cardiac chest pain, there are limitations. First, this is a single case report that demonstrated complete resolution of pain. This is not sufficient to make definitive conclusions regarding the benefits of early referral to a musculoskeletal specialist. Second, our patient had several risk factors to include current tobacco use, family history of early coronary disease, and recent deployment to Korea that would potentially warrant initial cardiopulmonary testing. In addition, the cardiac laboratory testing performed in the emergency room setting only included a troponin without other testing to evaluate risk such as a lipid panel. Finally, the active duty population has not been included in many studies evaluating testing and treatment of non-cardiac chest pain. Given the high-risk nature of this profession, more extensive cardiopulmonary testing may be warranted prior to referral to a musculoskeletal specialist.

## Conclusions

NCCP is a common diagnosis seen in both the emergency room and primary care settings. Despite exclusion of life-threatening conditions, low-risk patients with no significant risk factors often undergo advanced cardiovascular, pulmonary, or gastroenterology evaluations while only a small number of these patients are referred for a musculoskeletal evaluation. This current practice delays recovery of the patient and increases cost to the health care system. Our case represents a young active duty U.S. Marine with NCCP whose referral to an IPMC team was delayed by 12 months while undergoing initial cardiac testing. After being treated with osteopathic manipulation and a home exercise program consisting of strengthening and stretching, the patient had full resolution of chest pain. This case demonstrates the need for randomized trials that utilize early referrals to musculoskeletal providers for NCCP to determine the effect on time to recovery and health care costs.
